# On Your MARCKS…Get Set…Go: The Race to Explore Myristoylation in HF Is Underway∗

**DOI:** 10.1016/j.jacbts.2023.08.007

**Published:** 2023-10-23

**Authors:** Benjamin P. Keepers, Brian C. Jensen

**Affiliations:** a University of North Carolina School of Medicine, Chapel Hill, North Carolina, USA; b McAllister Heart Institute, University of North Carolina School of Medicine, Chapel Hill, North Carolina, USA; c Division of Cardiology, University of North Carolina School of Medicine, Chapel Hill, North Carolina, USA; d Department of Pharmacology, University of North Carolina School of Medicine, Chapel Hill, North Carolina, USA

**Keywords:** heart failure, gene therapy, cardiac remodeling, N-myristoylation, posttranslational modifications

**Figure:**
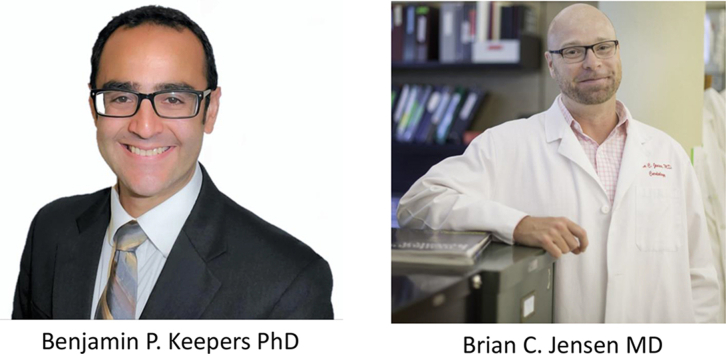

In this issue of *JACC: Basic to Translational Science*, Tomita et al^1^ make important contributions to our understanding of myristoylation in myocardial pathobiology in their article “Targeting N-myristoylation through NMT2 prevents cardiac hypertrophy and heart failure.” They investigate N-myristoyltransferase 2 (NMT2), one of 2 vertebrate homologs that catalyzes N-terminal myristoylation at Met-Gly motifs. In their opening figure, the authors demonstrate that NMT2 expression is reduced in both murine hearts subjected to transverse aortic constriction (TAC) and in failing human hearts. Furthermore, when mice subjected to TAC undergo abrogation of NMT2 expression by AAV9 RNAi, they suffer exacerbated fibrosis, cardiomyocyte hypertrophy, and mortality. Remarkably, AAV9-mediated overexpression of NMT2 significantly mitigates TAC-induced heart failure. Next, using a rigorous, Click-iT–based approach to quantify the myristoylated proteome in cardiomyocyte models in vitro, the authors deduce that cardiomyocyte exposure to either NMT2 RNAi or to angiotensin II reduces the levels of myristoylated alanine-rich C-kinase substrate (MARCKS), an actin-binding protein that transduces protein kinase C signaling.^2^ Through transgenic and pharmacologic dissection of the CAMK2-HDAC4 axis, they demonstrate that NMT2 inhibits CAMK2-dependent hypertrophy through MARCKS in a manner that correlates with reduced MARCKS membrane localization. In summary, the authors make a convincing argument that NMT2-mediated myristoylation suppresses pathologic cardiac remodeling at least in part through modulation of MARCKS expression and trafficking. These findings substantially advance our understanding of the contribution of myristoylation to heart failure pathobiology.

Myristoylation is a cotranslational modification that has a well studied role in protein membrane targeting and tertiary structure.^3^ It is a common (but not ubiquitous) feature of the proteome; approximately 1 in 50 proteins undergo posttranslational modification through NMT-mediated myristoylation. After the first 100 or so residues of the target protein have been translated and the initiating methionine residue has been removed, NMTs coordinate a nucleophilic attack by the amine group of the target’s N-terminal glycine on the thiol link between myristic acid (a saturated fat that derives its name from nutmeg, *Myristica fragrans*, from which it was isolated originally) and coenzyme A, resulting in attachment of the myristoyl group to the target and ejection of coenzyme A. Though it is an important membrane-targeting signal, myristoylation alone is usually insufficient to anchor proteins to the plasma membrane. Rather, it often is augmented by one of 3 types of potentiating forces: positively charged residues located proximal to the myristoylated glycine, palmitoylation of an adjacent cysteine residue, or interaction with another membrane-bound protein.

More than 3 decades ago, NMT1 was found to be critical to yeast viability, pointing to the ancient evolutionary origins of myristoylation as a mechanism of expanded protein functionality.^4^ Across Eukaryota, myristoylation is required for a variety of highly conserved proteins, such as ARFs, calcineurin, and AMPK. Though our understanding of the role of protein co- and posttranslational modifications in the pathogenesis of heart disease is relatively nascent, the importance of several types of modification has been established: succinylation, SUMOylation, neddylation, prenylation, acetylation, and glycation among them.^5^ Here, the authors add myristoylation to the growing list of protein modifications that play a critical role in cardiomyocyte function. They focus their mechanistic studies on MARCKS, though it is easily conceivable that myristoylation of other proteins might confer similar physiologic significance. For example, the structure and localization of the G_α_ subunit of G-protein coupled receptors is regulated by myristoylation, providing a very plausible connection between myristoylation and regulation of adrenergic signaling in cardiomyocytes.

It is also worth noting that the authors reasonably focused on NMT2 in their study after observing that NMT1 expression does not change in response to TAC. However, it remains possible that NMT1 and its targets have some physiologically significant role in the heart. In their supplemental data, the authors show that the myristoylation of dozens of proteins changes after RNA interference of NMT1 in H9c2 rat ventricular myoblasts, suggesting that the myristoylated proteome is responsive to NMT1. Though NMT1 expression levels are not changed by TAC, it is conceivable that pathologic insult to the heart may alter NMT1 enzymatic activity or substrate specificity. Because NMT1 and NMT2 are the only 2 known effectors of myristoylation—and much remains unknown about the functions of myristoylation in the heart—exploring NMT1’s function in cardiomyocytes seems warranted. To provide definitive and comparable evidence, future studies might utilize cardiomyocyte-specific gene traps to determine if NMT1 or NMT2 are required for murine myocardial function in physiologic and pathophysiologic contexts.

The use of well curated human samples in the present study significantly enhances its translational impact, scratching the surface of what we might learn by examining myristoylation in human heart failure. Interestingly, the ARIC study found that higher plasma levels of myristic acid are associated with more incident heart failure.^6^ Although the significance of that observation is uncertain (readers need not examine their consumption of nutmeg just yet), it suggests that the human myocardium may be responsive to circulating levels of myristate and that the cardiomyocyte myristoylated proteome may have a clinically significant role in heart failure.

In a hopeful conclusion to their paper, Tomita et al^1^ offer an intriguing new path toward a potential new heart failure therapeutic: augmenting NMT2 activity with small molecules or gene therapy to counter pathologic remodeling. Certainly, pharmacologically promoting enzyme activity is more difficult than inhibiting it, but it is not impossible. Moreover, adenoviral-based gene therapies that specifically target human cardiomyocytes are finally on the horizon, which means that gene transfer of NMT2 may be a viable option in the future.

Exciting discoveries undoubtedly lie ahead as the mysteries of myristic acid in the heart are unraveled further.

## Funding Support and Author Disclosures

The authors have reported that they have no relationships relevant to the contents of this paper to disclose.
